# High-intensity zones in dogs with lumbosacral intervertebral disc degeneration: insights from MRI and histopathological findings

**DOI:** 10.1080/01652176.2025.2486765

**Published:** 2025-04-07

**Authors:** S. Amir Kamali, Michelle Teunissen, Dirk Hendrik Nicolaas van den Broek, Elisabeth M. Burgers, Guy C. M. Grinwis, Keita Ito, Marianna A. Tryfonidou, Björn P. Meij

**Affiliations:** a Department Clinical Sciences, Faculty of Veterinary Medicine, Utrecht University, Utrecht, the Netherlands; b Department Biomolecular Health Sciences, Faculty of Veterinary Medicine, Utrecht University, Utrecht, the Netherlands; c Orthopedic Biomechanics, Dept. of Biomedical Engineering, Eindhoven University of Technology, Eindhoven, the Netherlands

**Keywords:** Annular fissures, disc herniation, decompression surgery, degenerative lumbosacral stenosis, lumbosacral pain

## Abstract

The diagnosis and management of lumbosacral pain in dogs is challenging, requiring thorough examination, with MRI playing a crucial diagnostic role. This retrospective study investigates the presence of high-intensity zones (HIZ) in the dorsal annulus fibrosus (AF) of the lumbosacral region on MRI and describes the corresponding histopathological features in dogs with intervertebral disc (IVD) degeneration. T2-weighted (T2W) and T1-weighted (T1W) sagittal MRI scans were evaluated using a classification system developed in human medicine to analyze HIZ characteristics. Among 836 dogs with IVD degeneration, 57 (6.8%) exhibited T2W HIZ, with a median age of 7 years and median weight of 33.7 kg. All cases with HIZ consistently exhibited radiological degenerative lumbosacral stenosis. The most common T2W HIZ shape was round (43%), while 14% of lesions also appeared hyperintense on T1W. Histopathological analysis of 11 dorsal AF samples collected during standard-of-care decompressive surgery revealed two patterns: reactive cystic structures (3/11) and granulation tissue (8/11), with differential MRI presentation. This is the first study to document HIZ in the lumbosacral level of dogs with IVD degeneration. With this recognition, prospective analyses and their correlation with clinical presentations will be essential in determining the role and prognostic significance of HIZ.

## 1. Introduction

Degenerative lumbosacral stenosis (DLSS) is a multifactorial disorder resulting from bone and soft tissue abnormalities that compress the nerve roots of the cauda equina in dogs. This compression leads to clinical signs like lumbosacral pain, lameness, and neuritis or radiculopathy with nerve root signs (Worth et al. 2019). The stenosis of the vertebral canal arises from various factors, including intervertebral disc (IVD) degeneration, ligament hypertrophy, osteophyte growth, and vertebral misalignment (Worth et al. 2019). Of these factors, IVD degeneration is one of the most common findings in dogs with DLSS (Meij and Bergknut 2010). Although IVD degeneration can have severe consequences, many dogs remain asymptomatic during its progression (Bergknut et al. 2013). Clinical signs typically emerge in the advanced stages of IVD degeneration, specifically when the disc either protrudes or extrudes into the spinal canal (herniation), compressing the spinal cord or nerve roots (Dou et al. 2021; Mohd Isa et al. 2022).

Magnetic resonance imaging (MRI) is widely regarded as the gold standard for diagnostic imaging in IVD degeneration (da Costa et al. 2020). However, the correlation between MRI findings, the clinical signs, and response to treatment in dogs with IVD degeneration is still not well understood (Kranenburg et al. 2013; Worth et al. 2019). For example, although histopathological IVD grades correlate with MRI grading, there is no significant correlation with pre-operative clinical signs (Kranenburg et al. 2013). Moreover, post-mortem spinal cord examinations of dogs without neurological signs revealed IVD protrusions in 40% of dogs older than seven years (Zani et al. 2018). Additionally, in working dogs that underwent surgical treatment for DLSS, no significant correlation was observed between imaging and postoperative outcomes (Worth et al. 2019). These findings highlight important concerns about the practicality and effectiveness of relying solely on MRI as a diagnostic tool for managing spinal disorders in veterinary cases, paralleling similar concerns in human medicine regarding low back pain (LBP) management (Bajpai et al. 2013; Latif et al. 2024).

The recently enhanced MRI quality in veterinary practice offers the potential to identify new imaging features associated with IVD degeneration. These imaging indicators could provide valuable tools to diagnose DLSS disease severity, guide clinical decision-making, and predict treatment outcomes. Notable examples include Modic changes (MCs) on MRI and endplate abnormalities on CT scan that require further studies in understanding the structural changes that may contribute to pain and neurological dysfunction in animals (Beukers et al. 2023; Tellegen et al. 2024).

Another promising but less-explored imaging finding in dogs is the high intensity zone (HIZ). The HIZ is an MRI finding characterized by a region of T2-weighted (T2W) high intensity within the annulus fibrosus (AF) of the IVD, suggesting the presence of an annular tear (Teraguchi et al. 2018). HIZs were first described in humans by Aprill and Bogduk in 1992 (Aprill and Bogduk 1992), who reported that these lesions were associated with symptomatic discs and correlated with clinical indicators of LBP. It has been suggested that the non-invasive nature of MRI for the identification of the HIZ as a marker for annular tears reduces the reliance on invasive discography for this purpose (Cheung and Luk 2019). However, subsequent studies suggested that the presence of HIZs does not consistently correlate with LBP, indicating that their role as a definitive marker of pain is still debated (Ricketson et al. 1996; Rankine et al. 1999; Carragee et al. 2000; Park et al. 2007; Takatalo et al. 2012; Wang et al. 2017).

Histological studies of HIZ in humans are limited and are completely absent in dogs. The histopathological evaluation of excised HIZ discs in humans has revealed the presence of disorganized granulation tissue (newly formed tissue rich in blood vessels with or without inflammatory cells) around AF tears raising the possibility that HIZ is caused by granulation tissue formed during the process of wound healing following annular tears (Peng et al. 2006). It has been shown that HIZ lesions are more common on the posterior side of the AF than on the anterior side (Teraguchi et al. 2016). The posterior portion of the AF is thinner and has fewer concentric lamellar layers to form lordotic curvature, potentially contributing to an asymmetric shape of IVD (Kumar and Pai 2020). This asymmetry exposes the posterior side of the AF to higher baseline stresses, accelerating the degenerative process, promoting tearing in this region and contributing to the formation of HIZ lesions (Peng et al. 2006). Despite these insights, the exact underlying causes and mechanisms of HIZ are still poorly understood.

Understanding the pathophysiology of HIZ is essential for determining its clinical importance in relation to back pain. To improve pathophysiological understanding, Teraguchi et al. introduced a classification system for the T2W MRI imaging of HIZ, including morphology, location and intensity of HIZ (Teraguchi et al. 2016). However, its relationship with IVD degeneration remains unclear (Teraguchi et al. 2016). This leaves a significant gap in our understanding of the pathophysiological basis underlying HIZ MRI findings.

Therefore, this study describes the prevalence of dorsal HIZ at the lumbosacral level in dogs and categorizes them using the classification system developed by Teraguchi et al. (Teraguchi et al. 2016). Furthermore, in the available subset of samples derived from AF tissue with HIZ, histopathological analysis was performed to provide an insight into ­tissue characteristics associated with the HIZ observed on MRI.

## 2. Materials and methods

### 2.1. Data collection

Inclusion criteria were dogs referred to the Companion Animal Clinic at Utrecht University between 2013 and 2023 with lumbosacral MRI scans. Exclusion criteria included the absence of a HIZ in the dorsal AF at the L7-S1 level, the absence of a T2-weighted image sequence (sagittal and transverse views) on the MRI scans, or the presence of other conditions such as acute discospondylitis, acute trauma, or neoplasia that could interfere with the MRI signal or histopathological findings of AF tissue biopsy.

The medical records of included cases were analyzed for information on signalment (age, breed, and gender), and histopathological findings of dorsal AF tissue harvested during decompression surgery. Tissue samples were retrieved from the pathology archives for further evaluation. Additionally, written MRI reports were examined for evidence of DLSS, based on the descriptive MRI findings commonly associated with this disorder, as previously detailed (Wiersema et al. 2021). These findings included IVD degeneration (Pfirrmann grade (PG) (Lee et al. 2021)), disc protrusion at L7-S1 graded as <25% or ≥25% stenosis of the spinal canal, presence of MCs (Beukers et al. 2023), swelling of the spinal nerves, intervertebral foraminal stenosis, pressure on the cauda equina, and facet joint degenerative disease.

### 2.2. Magnetic resonance imaging (MRI)

Lumbosacral MRI studies were performed on dogs under general anesthesia in dorsal recumbency with a 1.5-Tesla magnet (Philips Ingenia, Eindhoven, the Netherlands). Sagittal T2W fast-spin echo (FSE) sequences (TR 3000 ms, TE 120 ms, slice thickness 5 mm, FOV 270 mm x 270 mm) and sagittal T1W sequences (TR 540 ms, TE 10 ms, slice thickness 5 mm, FOV 270 mm x 270 mm) were acquired. In a subset of dogs, post-contrast sagittal T1W images were acquired 1 min after intravenous administration of gadoterate meglumine (0.15 mmol/kg, Dotarem 0.5 mmol/mL, Guerbet, Roissy CdG Cedex, France).

In cases where the initial reviewer (S.A.K.) suspected HIZ, confirmation was sought *via* a consensus judgment involving a board-certified veterinary radiologist (D.H.N.B.). Following that, all MRI studies with HIZ were graded by two observers, a board-certified veterinary radiologist (D.H.N.B.) of the European College of Veterinary Diagnostic Imaging (ECVDI) and first-year ECVDI resident (E.B.) using a modified version of the previously proposed MRI classification system by Teraguchi et al. (Teraguchi et al. 2016) for the HIZ shape on T2W and signal intensity on T1W sequences. In this modification, the intensity of the HIZ on T1W sequences was compared to the intensity of the AF of a non-degenerative disc within the same patient rather than using the vertebral body bone marrow. Furthermore, the presence of other spinal MRI phenotypes such as MCs (Beukers et al. 2023) and Schmorl’s node were evaluated, as well as disc degeneration, which was graded using the modified Pfirrmann grading system for dogs (Lee et al. 2021). Additionally, when available, the morphology of the HIZ during flexion, and in follow-up MRI images was analyzed to assess changes in the HIZ across different positions and over time, respectively. To ensure accurate identification of HIZ on MRI during consecutive follow-up time points, anatomical landmarks were identified, and a detailed initial assessment was conducted. Consistent MRI sequences were employed across all follow-up scans to maintain comparability and minimize variability. Furthermore, a thorough initial assessment of the HIZ—including its location, shape, and characteristics—served as a crucial reference for detecting any changes over time.

The study evaluated the classification of HIZ shapes on T2W images and the reliability of categorizing the T1W HIZ signal as ‘hypointense’, ‘isointense’, or ‘hyperintense’ based on Table 1.

**Table 1.** MRI assessment of lumbosacral high intensity zone (HIZ) on T2-weighted (T2W) and T1W MRI scans.

Variables	Definition
*Shape of HIZ on T2W image*	
Round	Concentric or oval cavity
Fissure	Parallel and transverse layer to the adjacent endplate
Vertical	Vertical layer to the adjacent endplate
Rim	Oblique radiating layer from the adjacent endplate
Enlarged	Greater concentric area than typical round HIZ
Signal type of HIZ on T1W image*	
T1W low-intensity type of HIZ	Decreased signal than the adjacent normal AF tissue on T1W sagittal MRI
T1W high-intensity type of HIZ	Increased signal than the adjacent normal AF tissue on T1W sagittal MRI
T1W iso-intensity type of HIZ	Same signal as the adjacent normal AF tissue on T1W sagittal MRI

Classification of HIZ based on Teraguchi et al. (Teraguchi et al. 2016) with modification (*): the intensity of the HIZ on T1W sequences was compared to the intensity of the AF of an adjacent non-degenerative disc instead of the vertebral bone marrow. MRI: magnetic resonance imaging, AF: annulus fibrosus.

### 2.3. Histopathology and immunohistochemistry (IHC) analysis

From the animals included in this study in which a HIZ was identified and that underwent standard-of-care dorsal laminectomy and partial microdiscectomy of L7-S1 level by a board-certified veterinary surgeon (B.M.), surgical samples were collected from the dorsal AF during dorsal fenestration of L7-S1 IVD in a subset of animals. The precise type and location of IVD herniation were defined based on the surgical records, including the location of disc protrusion, as well as the site and extent of resection. In the most recently harvested samples from cases included in this study, the records were further supported by a schematic drawing of the surgical sample aiding in the histopathological identification of dorsal-ventral, cranial-caudal, and right-left orientations. These samples were inked by the surgeon. The harvested AF tissues were formalin-fixed (Formaldehyde 4%, buffered, Klinipath B.V., the Netherlands). Four µm thick hematoxylin and eosin (H&E) sections were evaluated by two veterinary pathologists (G.G. & S.A.K.) using The Olympus BX45 light microscope.

Based on the phenotype of the lesions within the AF, additional tissue sections were prepared for immunohistochemical analysis. Briefly, consecutive tissue sections were used for immunochemical staining for IBA1 (marker for histiocytic cells; rabbit α IBA-1, Fujifilm WAKO Company, Osaka, Japan; Cat.#019-19741), Factor VIII (marker for endothelial cells; rabbit α Factor VIII, Dako Co., Copenhagen, Denmark; used at 1/500, Cat.#A0082) and cytokeratin AE1/AE3 (epithelial cells; Rabbit α cytokeratin AE1/AE3, Dako Co., Copenhagen, Denmark; Cat.#M3515). The 4 µm paraffin sections were dewaxed, hydrated through a series of ethanol solutions (100%, 95%, 80%, and 70%), and rinsed twice with Aquadest for 3 min each. Following microwave antigen retrieval for cytokeratin AE1/AE3 and IBA1 marker or enzymatic retrieval (0,1% pronase) for Factor VIII, the sections were rinsed with PBS, three times for 5 min each. Next, 1% H2O2 in methanol was used in order to block the endogenous peroxidase activity. The samples were then incubated with a blocking solution (1% bovine serum albumin) for 20 min at room temperature. The tissue sections were then incubated overnight at 4 °C with rabbit IBA-1 antibody (used at 1/1000), rabbit Factor VIII (used at 1/500) and rabbit cytokeratin AE1/AE3 antibody (used at 1/1600). After washing with PBS/tween, secondary antibodies (1:200 biotin-labeled goat anti rabbit IgG) were applied for 30 min at room temperature. Each step was followed by a PBS rinse for 5 min, repeated three times. Finally, tissue sections were counterstained with hematoxylin and mounted with Aquatex^®^ (Sigmaaldrich, 108562). DAB-positive cells for each marker were automatically identified using positive cell detection in QuPath software (by S.A.K.) (Bankhead et al. 2017). Subsequently, hotspot areas were identified using a semi-automated method by generating density maps in QuPath, with a smoothed nucleus DAB optical density mean of 50 μm. The hotspot areas for different markers were then evaluated by expanding cosine vector similarity to a matrix which allowed for an assessment of their relationships across the markers (Afshari and Tizhoosh 2021). The localization of immunopositive cells within the hotspot map for the IBA1 and Factor VIII markers was analyzed using VGG16, a deep convolutional neural network model developed by the Visual Geometry Group (VGG) at the University of Oxford.

## 3. Results

### 3.1. Demographics and MRI diagnosis

**Figure 1.**
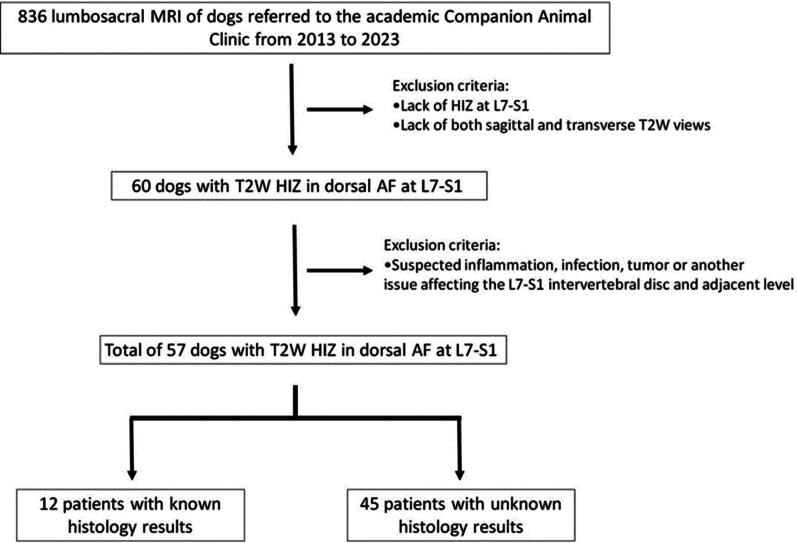
Study design for retrospective case selection of dogs with high intensity zone (HIZ) in the dorsal annulus fibrosus (AF) on T2-weighted (T2W) MRI.

Between 2013 and 2023, 838 dogs underwent MRI of the lumbosacral spine at the Companion Animal Clinic at Utrecht University. A total of 776/838 cases were excluded due to the absence of a HIZ in the dorsal AF of L7-S1 or due to the absence of both sagittal and transverse T2W images. Of the remaining 60 cases, 3 were excluded due to suspected discospondylitis, neoplasm, or other issues affecting the L7-S1 intervertebral disc (IVD) and adjacent levels (Figure 1).

Ultimately, 57 of the 838 patients, consisting of 33 males (60% neutered) and 24 females (54% neutered), were included in the study. At the time of the MR imaging, the dogs ranged in age from 1 to 12 years old (median: 7 years old) and weighed 9.5 to 52 kg (median: 34 kg). The weight in 5 dogs (2 male and 3 female) is unknown. All but 1 of the included patients were classified as non-chondrodystrophic (NCD) with 48 (84%) purebred and 8 (14%) mixed breed dogs. The breed of 1 dog was unknown. Included purebred dogs were German Shepherd Dog (*n* = 8), Labrador retriever (*n* = 7), Bernese Mountain Dog (*n* = 3), Border Collie (*n* = 3), Weimaraner (*n* = 3), Labradoodle (*n* = 2), Rhodesian ridgeback (*n* = 2), Alaskan Malamute (*n* = 1), Australian Labradoodle (*n* = 1), Belgian Shepherd Dog (*n* = 1), Boxer (*n* = 1), Dutch Partridge Dog (*n* = 1), English Cocker Spaniel (*n* = 1), Flatcoated retriever (*n* = 1), French Bulldog (*n* = 1), Golden Retriever (*n* = 1), Goldendoodle (*n* = 1), Long-Haired Collie (*n* = 1), Nova Scotia Duck Tolling Retriever (*n* = 1), Pitbull Terrier (*n* = 1), Portuguese Sheepdog (*n* = 1), Rottweiler (*n* = 1), Slovakian wirehaired pointer (*n* = 1), Stabyhoun (*n* = 1), Tosa (*n* = 1), White Swiss Shepherd Dog (*n* = 1) and Wirehaired Pointing Griffon (*n* = 1). Of the 57 dogs included in this study, 19 underwent decompression surgery (dorsal laminectomy) following the initial MRI that revealed the HIZ, 34 had no history of surgery, and the surgical history of 4 dogs was unknown. All dogs showed characteristics of DLSS, as indicated by the MRI findings described in the M&M section.

### 3.2. High intensity zone on MRI

**Figure 2.**
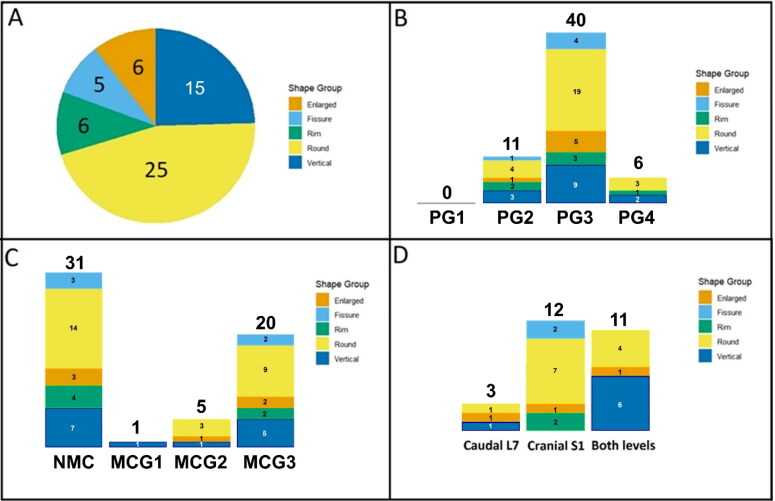
Overview of high intensity zone (HIZ) on MRI in dog patients with lumbosacral intervertebral disc degeneration, A: HIZ shape morphology (%), B: Pfirrmann grading (PG) of the L7-S1 level, PG1: grade 1, PG2: grade 2, PG3: grade 3, PG4: grade 4, C: Modic change grading, NMC: no modic change, G1: grade 1, G2: grade 2, G3: grade 3, D: Modic change in caudal L7 endplate, cranial S1 endplate, or both levels the numbers within each color section in panel a indicate the quantity of each identified HIZ shape. Numbers displayed above the bars in panels B-D indicate the patient numbers.

**Figure 3.**
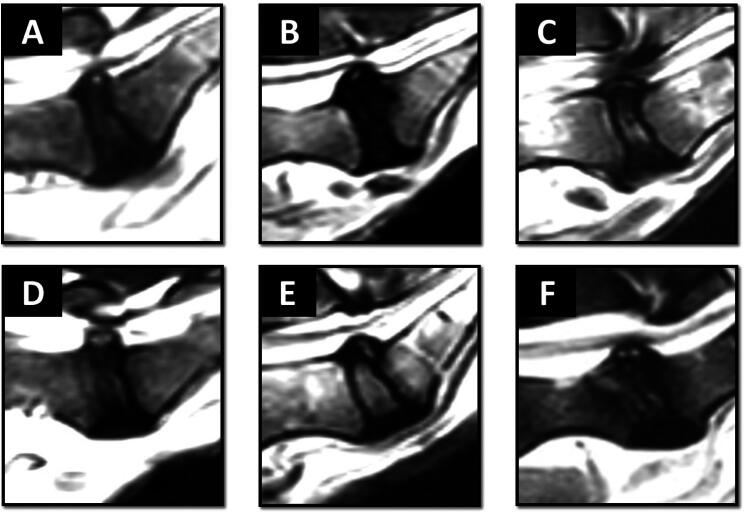
Sagittal T2W MRI scans depicting varied morphologies of the high intensity zone (HIZ) in the lumbosacral area (L7-S1 level). The HIZ forms were classified as round (a), rim (B), vertical (C), enlarged (D), and fissure (E) types. Single or double HIZ may be present in the same disc, e.g. double round HIZ (F).

The modified Teraguchi et al. (Teraguchi et al. 2016) classification system was used for the 57 dogs to determine the shape of HIZ in the T2W sequence as well as the intensity of the HIZ in T1W MRI. The most prevalent shape was round (25/57), followed by vertical (15/57), rim (6/57), enlarged (6/57) and fissure shapes (5/57) (Figures 2A and 3).

This study also assessed IVD degeneration and the presence of MCs in the bony endplate. Most patients with HIZ were classified as PG 3 (40/57), and included enlarged (19/40), fissure (5/40), rim (3/40), round (9/40), and vertical (4/40) HIZ shapes. Among patients with HIZ, 11 fell into PG 2 classification, with shapes distributed as enlarged (4/11), fissure (1/11), rim (2/11), round (3/11), and vertical (1/11). Six patients were categorized as PG4, characterized by enlarged (3/6), fissure (2/6), and round (1/6) shapes, while rim and vertical shapes were absent (Figure 2B). We found that 45% (26/57) of the dogs exhibited MCs. Specifically, 1 had grade I, 5 had grade II, and 20 had grade III (Figure 2C). The location of MCs was either at the caudal L7 endplate, S1 cranial endplate, or both (Figure 2D). The prevalence of various HIZ shapes across Modic change grades (MCG) and their localization at specific spinal levels is also illustrated in Figures 2D and 2C. Schmorl’s nodes were not observed.

#### 3.2.1. Intensity of HIZ on the T1W sequence

**Figure 4.**
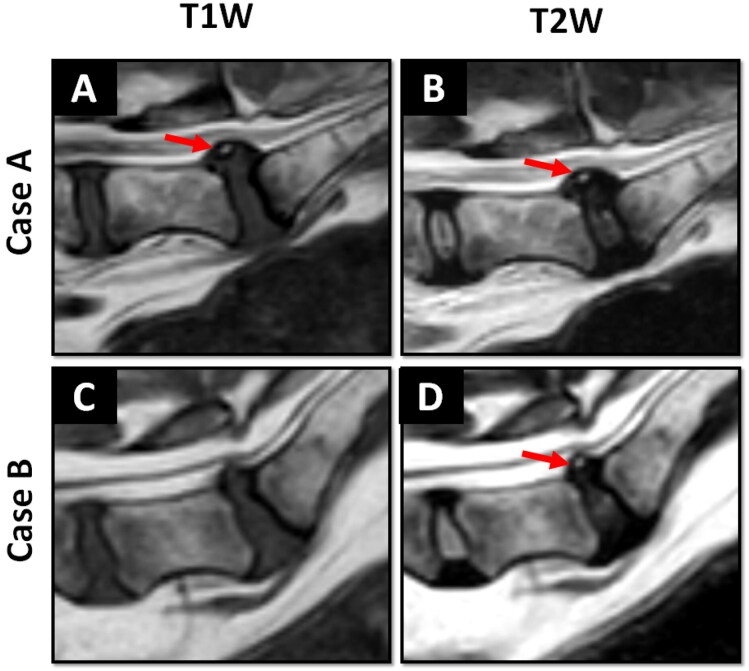
Sagittal plane MRI scans showing the detection of the L7-S1 high intensity zone (HIZ) on T2-weighted (T2W) images (B and D) and the T1W images (a and C). In this investigation, the L7-S1 HIZ appeared hyperintense (case A) or isointense (case B) on T1W imaging when compared to the adjacent L6-L7 normal annulus fibrosus tissue on T1W. Red arrows: HIZ.

The intensity of HIZ on T1W at L7-S1 level was evaluated and compared to the AF of an adjacent non-degenerative disc in all included patients except for one, where the T1 sequence and related data was not available in the database. Of the observed HIZs, 86% (48/56) appeared isointense, 14% (8/56) appeared hyperintense and none were hypointense (Figure 4).

#### 3.2.2. Visualization of HIZ in contrast-enhanced T1W MRI

**Figure 5.**
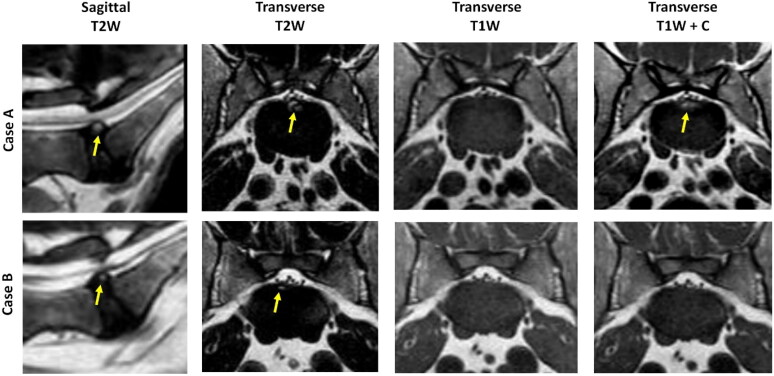
The examination of high intensity zone (HIZ) at the L7-S1 level using contrast media indicated that T2W HIZ was apparent on contrast-enhanced T1W (T1W + C) images in 3 out of 21 cases presenting iso-intense on non-contrast T1W (illustrated by case A). In the 18 out of 21 patients, despite the use of contrast media, HIZ was not detected on T1W images (illustrated by case B). Yellow arrows indicate HIZ.

The existence of HIZ at the L7-S1 level in T1W was also assessed, using contrast agent (gadolinium) to increase the T1W signal intensity (available in 22 patients). When using contrast media, the HIZ became visible in 3 out of 22 cases where HIZ appeared isointense in non-contrast T1W. HIZ was not detectable in contrast-enhanced T1W images in the majority (19/22) of cases (Figure 5).

#### 3.2.3. Presence of HIZ in different positioning and consecutive MRI scans

**Figure 6.**
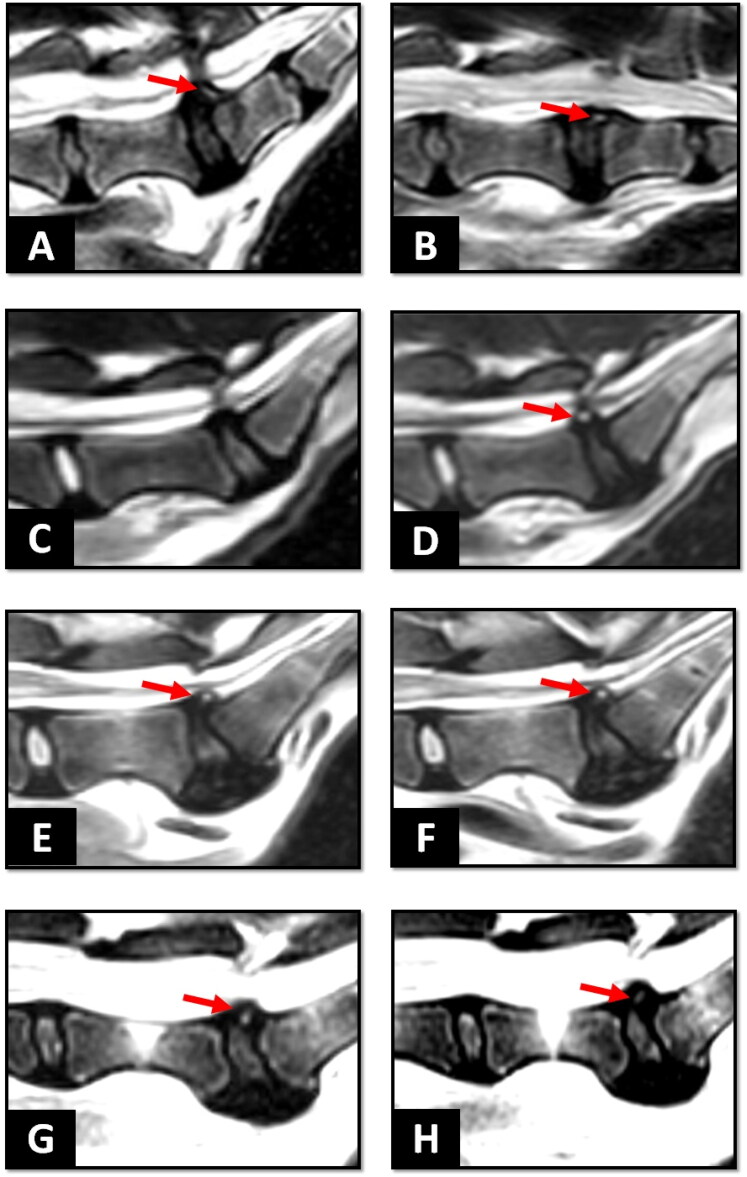
Presence of high intensity zone (HIZ) in dynamic and consecutive MRI scans in dogs with intervertebral disc degeneration. Dynamic MRI showed change in shape of HIZ with the spine in extension (A) and flexion (B) positions. Evaluation of HIZ progression in consecutive MRI scans: in one dog with disc protrusion HIZ was initially absent in the dorsal annulus fibrosus (C) but detected 3 years later (D). It should be noted that the disc also displayed a decrease in signal intensity, which signifies ongoing disc degeneration. In another dog, HIZ remained unchanged in shape and location on MRI scans with 1 year interval (E, F). Additionally, HIZ was observed to resolve in another dog within a 2.5-month follow-up period (G, H). Red arrows indicate HIZ.

Dynamic MRI with sagittal T2W sequences acquired in extended and flexed positions were available for nine dog patients with T2W HIZ. In these patients, variations in HIZ shape and localization were apparent (Figure 6A–B).

Additionally, ten dogs had multiple lumbosacral MRI studies at varying intervals (2.5 to 16 months), which demonstrated that HIZ could appear later (2/10) (Figure 6C-D), remain unchanged (6/10) (Figure 6E–F), or even resolve over time (2/10) (Figure 6G–H). A data sheet containing the information of the 57 included animal patients, including their signalments, surgical history (dorsal laminectomy) and associated MRI findings, is provided in the supplementary Excel file.

### 3.3. Histopathology and immunohistochemistry (IHC) of HIZ

**Figure 7.**
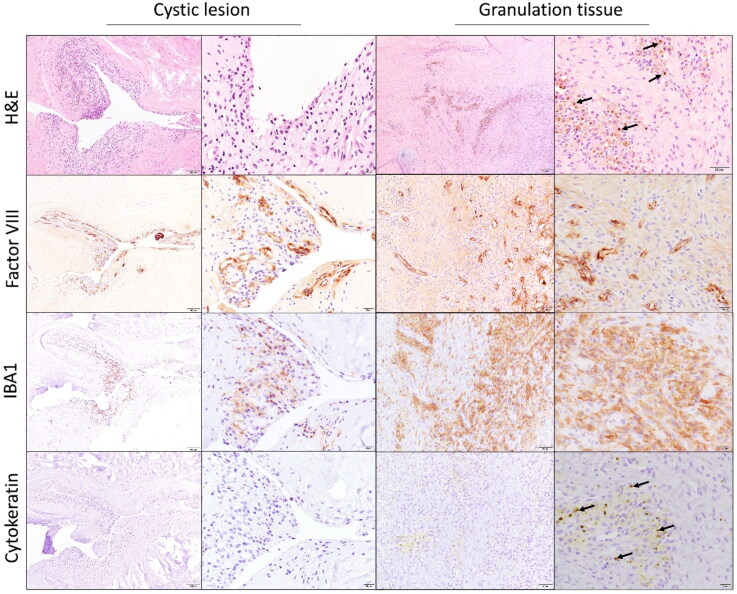
Micrographs of two distinct histopathologic presentations of high-intensity zones (HIZs). The inflamed cystic structure is surrounded by numerous mononuclear cells and newly formed blood vessels. There are many macrophages with hemosiderin pigment (black arrows) in the granulation tissue. Factor VIII, an endothelial marker, is positive in both histopathological presentations whereas the epithelial marker cytokeratin is undetectable. H&E: Hematoxylin and eosin, and IBA1: ionized calcium-binding adapter molecule 1.

Out of the 19 patients who underwent decompression surgery, 12 patients had samples sent for histopathological analysis. The histopathological sample taken from one of the patients was excluded from the study due to the unclear origin of the biopsy. Histopathological examination revealed two distinct histological presentations of the HIZ area within the AF tissue. The first histological presentation was a highly vascularized fibrous tissue (granulation tissue; 8 out of 11 cases). The second was an extensive annular tear in the AF tissue surrounded by fibrovascular tissue, resembling cystic lesions (3 of 11 cases). These cystic formations were surrounded by a variable number of round cells (mainly lymphocytes, plasma cells and macrophages; Figure 7). Additional immunohistochemical analysis was conducted to characterize the phenotype of these cells. Immunopositivity for IBA1, a macrophage marker, was observed in both histological conditions—granulation tissue and cyst-like structures. The granulation tissue had a higher proportion of IBA1-positive cells which also contained brown hemosiderin pigment indicative of prior hemorrhage in the vicinity of the lesion (Figure 7). However, only some of the infiltrating round cells surrounding the cystic formations were positive for the IBA1 marker. Additionally, both histologic presentations showed immunopositivity for Factor VIII, an endothelial marker. Its expression was particularly noticeable around the cystic structures, where newly formed blood vessels were evident. Notably, neither the granulation-type lesion nor the cells lining the cystic formations showed the epithelial marker cytokeratin, indicating a lack of epithelial differentiation in these lesions.

**Figure 8.**
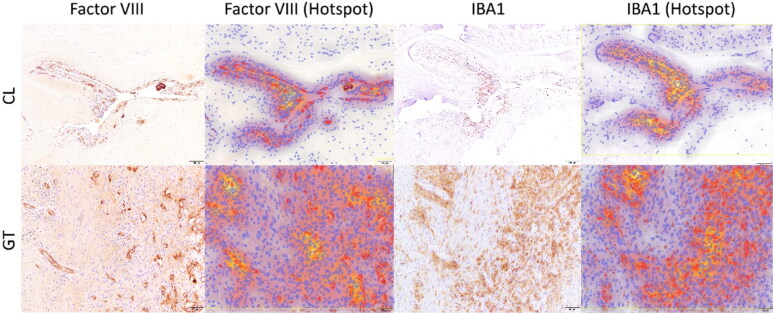
The relationship between Factor VIII and the IBA1 marker was assessed using density mapping in QuPath software. This analysis revealed a close similarity between the hotspot areas, indicating a strong correlation between the presence of new blood vessels (Factor VIII) and the infiltration of macrophages (IBA1-positive cells).

The relationship between Factor VIII and the IBA1 marker was assessed using the density map feature of QuPath software. The hotspot regions identified through this analysis revealed a close spatial relationship between IBA1-positive cells (histiocytes) and Factor VIII-positive cells (indicative of new blood vessel formation) which is indicating that regions with high histiocytic activity also displayed increased blood vessel formation (Figure 8).

### 3.4. Histopathological findings and the shape of HIZ on MRI

**Figure 9.**
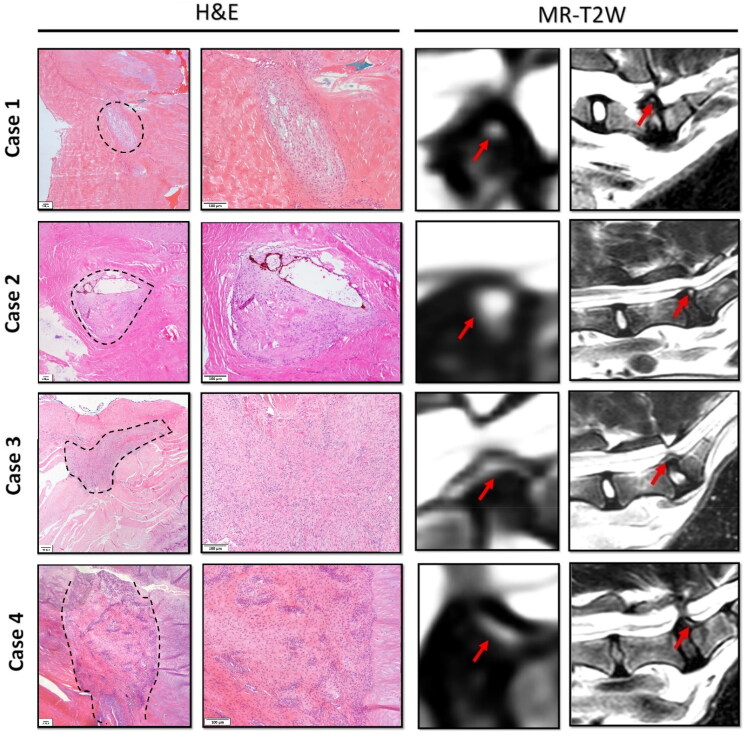
Comparison of high intensity zone (HIZ) shapes in histopathology and corresponding T2W MRI. Cases 1 and 2 display round-shaped HIZs, as defined by Teraguchi et al. (Teraguchi et al. 2016), corresponding to round granulation tissue and cystic lesions (black dotted line) on histology. Case 3 involves a patient with a vertical (linear) HIZ, with histology confirming linear granulation tissue, while case 4 depicts a rim-shaped HIZ that aligns with the orientation of granulation tissue in histology, indicated by the black dotted lines. Red arrows denote the HIZ.

In four recent cases which were included prospectively in this study, tissue inks were utilized to mark the sides of the excised AF tissues to ensure proper topographic orientation within the paraffin blocks and correlate MRI findings with histopathology. The shapes observed in the MRI images were consistent with the histopathological findings. For instance, in the cases of a round HIZ on T2W MRI, histopathology revealed either granulation tissue or a round-shaped cystic lesion (cases 1 and 2, respectively). In another case, a vertical (linear) shaped HIZ on T2W MRI corresponded with a vertical granulation tissue pattern (case 3). Similarly, a rim-shaped HIZ matched the granulation tissue shape observed in the MRI (case 4). Interestingly, in T2W MRI (cases 1 and 2), the round HIZ linked to granulation tissue showed lower intensity than the HIZ linked to inflammatory cystic lesions. Furthermore, T2W imaging showed an intensity gradient in the inflamed cystic lesion, with a higher intensity seen in the dorsal region where the hollow of the AF tears—which is probably filled with fluid—and a decreasing intensity in the ventral region, where proliferating mesenchymal cells and mononuclear inflammatory cells were seen (Figure 9). The clinical signs and corresponding MRI findings along with histopathology results from these eleven patients who underwent decompression surgery are listed in supplementary Table S1.

## 4. Discussion

The incidence of HIZ in the posterior AF has been observed in both symptomatic and asymptomatic human patients since it was first reported by Aprill and Bogduk in 1992 (Aprill and Bogduk 1992). However, HIZ has not yet been formally recognized in veterinary terminology and remains poorly understood. To the authors’ knowledge, this is the first study to retrospectively investigate MRI-detected HIZ morphology and its histopathological characteristics in dogs, focusing on lesions in the dorsal AF that resemble the human posterior AF (Carragee et al. 2000; Lam et al. 2000; Chen et al. 2011; Teraguchi et al. 2016), and this was achieved by capturing the topographic orientation during surgical collection of AF tissue specimens.

To investigate the presence of the HIZ in the dorsal AF detected on T2W imaging, we characterized HIZ on two key components; shape and T1W intensity, following a modified classification system based on Teraguchi et al. (Teraguchi et al. 2016). Our findings demonstrated that the ‘round’ shape of HIZ was the most prevalent shape in dogs, accounting for 43% of cases (25 out of 57 cases) at the L7-S1 level. While no correlation with clinical data was assessed in the present study, these observations suggest that the ‘round’ shape may have pathological importance and emphasize the need for additional investigation into its clinical implications for veterinary patients. Furthermore, the finding that larger HIZs on consecutive sagittal T2W images are more consistent indicators of pain highlights the necessity of improving shape definitions and imaging methodologies in future study (Wang and Hu 2021).

The second key component for characterizing HIZ in this study was T1W intensity of the HIZ detected on T2W imaging. We modified the original classification system by comparing the T1W intensity of HIZ to adjacent healthy AF tissue rather than bone marrow, to reduce variation from fat content and other pathologies like MCs (Beukers et al. 2023; Mourad et al. 2023). Many substances such as contrast agents, methemoglobin, melanin, lipids, minerals, some proteins or mucins, etc. may exhibit a high T1W signal intensity (Peyrot et al. 2015; Gupta et al. 2017). Dual HIZ (T2W and T1W imaging showing high intensity) in human patients was associated with calcification or bony fragments within the AF tissue, while absence of high intensity signal on T1W was associated with fissures and granulation tissue (Shan et al. 2017; Nguyen et al. 2022). Although our investigation found dual HIZ patterns in dogs, we lacked tissue samples to conduct a thorough histological analysis of these lesions. Another proposed explanation for dual HIZ is that these patterns represent different stages of the pathological process, such as fluid accumulation, neovascularization, and healing annular tears (Teraguchi et al. 2016). Similar trends were observed in dogs, where HIZ progression in follow-up imaging appeared to be influenced by the underlying pathology. It could be hypothesized that small annular fissures might evolve into less visible scar tissue, while extensive annular tears could develop into persistent fluid-filled cystic lesions, contributing to chronic pain. This aligns with the well-known complexity of HIZ evolution in discogenic pain, where certain HIZs are shown to persist over time while others may change or resolve over time (Mitra et al. 2004). The mechanisms of dual HIZs as well as the discrepancies between imaging and pathology have been documented here but cannot be fully understood without further histological studies on humans and dogs.

The histological study is crucial in understanding the pathophysiology of HIZs detected by MRI. Histology helps explain the nature of inflammation and the degenerative process depicted in these MRI results. Previous research in humans has suggested that HIZs may be associated with fluid or mucoid components in annular tears, although this has yet to be confirmed by histopathology (Yu et al. 1989). Furthermore, the herniating nucleus pulposus trapped within the ruptured lamellae of AF tissue may also cause inflammation and edema resulting in the typical bright signal observed on MRI (Weidenbaum et al. 1992; Jha et al. 2016). Peng et al. (2006) conducted the first histological analysis of HIZs in eleven human patients. In individuals with low back pain, HIZs indicated granulation tissue ingrowth into annular tears in the posterior section of the affected disc (Peng et al. 2006). Dongfeng et al. (1976) further characterized the HIZs as containing abundant number of proliferating reactive fibroblasts, small round cells and neovascularization. Within the HIZs, significantly more TNF and CD68 positive cells, both histiocytic markers, were present than in control samples (Dongfeng et al. 1976). In line with this, in the present retrospective dog study, we observed granulation tissue in the majority of HIZ lesions containing numerous blood vessels positive for Factor VIII, as well as numerous inflammatory cells, the majority of which were histiocytes positive for IBA1. Another notable histopathological presentation in this study was the presence of reactive cystic lesions. We postulate that these represent annular tears, characterized by neovascularization, infiltration of round cells and inflammatory cell spreading along their edges. However, the round cells lining the margins of these cystic lesions expressed no cytokeratin indicating that they did not represent synovial or epithelial cysts. Interestingly, HIZ linked to reactive cystic lesions showed higher signal intensity than HIZ associated with granulation tissue. The reason for this variation could be that the granulation tissue has a lower fluid content than the inflammatory cystic lesions. The quantity and size of blood vessels, the degree of inflammation and subsequent edema in annular lesions—a phenomena also documented in earlier research—are likely to have an impact on the intensity of HIZ (Peng et al. 2006).

To better understand the underlying causes of HIZs in symptomatic patients, advanced imaging techniques should be employed in veterinary cases. For instance, enhanced gadolinium uptake in HIZ regions, indicative of granulation tissue or inflammation-induced neovascularization, has been observed in the current retrospective study of dogs and reported in previous clinical studies (Bartynski et al., 2023). The difference between HIZs that are visible versus those that are non-visible on contrast-enhanced T1-weighted images remains to be fully elucidated in veterinary cases.

The clinical importance of HIZ in veterinary cases remains under discussion, as they could be incidental findings. However, the relevance of HIZs to discogenic pain and intervertebral disc degeneration is well-supported by previous literature on human patients (Carragee et al. 2000; Lam et al. 2000; Chen et al. 2011; Teraguchi et al. 2016). Consistent with prior research, our study found that over 70% of dogs had a Pfirrmann grade 3, and nearly half exhibited MCs at the L7-S1 level. These findings confirm the relationship of HIZs with increased rate of degenerative changes, underlining their clinical significance, and the importance of taking degenerative changes into account when assessing these lesions. Overall, the results of this study enrich notions of HIZ in dogs and reveal similarities to human cases, which open potential avenues of comparative research.

### Limitations

Major limitations are the retrospective design of the study and the lack of an evidence-based questionnaire for clinical signs and outcome, as well as a more standardized categorization system, specifically regarding the shape of HIZs. In addition, other limitations such as the presence of concurrent painful conditions, such as degenerative joint disease in other joints (e.g. the knee joint), disc herniation or disc protrusion at other distant levels of the affected intervertebral disc, complicate the identification of the origin of the clinical signs. Furthermore, two of the patients were external referrals for MRI scans at our center, and the detailed clinical signs or information regarding other painful conditions in these two animals were not available. These factors presented the major challenges for inclusion of clinical signs in this study.

Moreover, the relatively low number of samples available for histopathological analysis does not allow to form conclusions with respect to the two histological HIZ subtypes observed. Furthermore, this study lacks histopathology findings for the cases that present as hyperintense in T1W, which would be useful in determining the phenotype of the lesion in the dorsal AF. One of the most interesting aspects not fully addressed in this study is the correlation between distinct histopathological conditions (cystic structures and granulation tissues) and their visibility on contrast-enhanced MRI images. However, due to the limited number of AF samples collected, it is not possible to draw conclusions or discuss this aspect in the present study. Future research on this type of tissue sample may shed more light on the nature of HIZ and its relationship to clinical signs.

In this context, It is recommended to carry out a prospective study using control groups, including healthy dogs as positive controls and dogs with DLSS diagnoses as negative controls. This method will enable comparative analysis and minimize the possibility of bias.

## Conclusion

This study is the first to categorize HIZs detected on T2W imaging in the lumbosacral region of dog patients and introduces a modified imaging strategy using T1W MRI to assess HIZ variations. We highlight the relationship between HIZs on MRI and histological findings in dogs with lumbosacral IVD degeneration and identify two major histological subtypes involving granulation tissue and inflamed cystic lesions. Hypothetically, there is a chance that these two histological presentations may be related, as the fluid from AF tears (cystic lesions) might resorb over time and possibly change into granulation tissue throughout the healing process. The modified classification of HIZs serves as a platform for future prospective research to ultimately understand the clinical relevance of HIZs in the veterinary field.

## Supplementary Material

Supplemental Material
