# Supplementary material for: High-intensity zones in dogs with lumbosacral intervertebral disc degeneration: insights from MRI and histopathological findings
Source: Vet Q. 2025 Apr 7;45(1):1–13. doi: 10.1080/01652176.2025.2486765 (PMC11980243; doi:10.1080/01652176.2025.2486765)
Supplement: Supplemental Material [file TVEQ_A_2486765_SM9202.zip › Suppl/Supplementary Table (4).docx]

**Supplementary Table 1.** Clinical signs and corresponding MRI findings in 11 patients who underwent decompression surgery, along with histopathology results

| No. | Breed | Low back pain | Lordosis test | LS pressure test | Stiffness | Uni/Bi-lateral Lameness | Shape on MRI | Contrast enhancement on T1W | Intensity on T1W | Histopathology |
| --- | --- | --- | --- | --- | --- | --- | --- | --- | --- | --- |
| 1 | Australian labradoodle | Yes | Positive | Positive | Yes | Yes (Right) | Vertical | NV | Isointense | Granulation tissue |
| 2 | Weimaraner | Yes | Positive | Positive | Yes | Yes (Bilateral) | Round | NV | Isointense | Granulation tissue |
| 3 | Labrador Retriever | Yes | Positive (Right) | Positive | No | No | Round | V | Isointense | Cystic lesion |
| 4 | Mix | Yes | Negative | Positive | No | No | Fissure | n/a | Isointense | Cystic lesion |
| 5 | French bulldog | Yes | Positive | Negative | Yes | Yes (Right) | Round | NV | Isointense | Granulation tissue |
| 6 | English Cocker Spaniel | Yes | n/a | Negative | Yes | Yes (Bilateral) | Enlarged | n/a | Isointense | Granulation tissue |
| 7 | Wirehaired Pointing Griffon | Yes | Positive | Positive | Yes | No | Enlarged | n/a | Isointense | Granulation tissue |
| 8 | Labradoodle | Yes | n/a | Positive | n/a | Yes (Right) | Rim | NV | Isointense | Granulation tissue |
| 9 | Border collie | Yes | Positive | Positive | No | No | Rim | n/a | n/a | Granulation tissue |
| 10 | Slovakian wirehaired pointer | Yes | n/a | Positive | Yes | Yes (Right) | Vertical | n/a | Isointense | Cystic lesion |
| 11 | German Shepherd | Yes | Positive | Positive | No | Yes (Right) | Rim | NV | Isointense | Granulation tissue |

n/a: Not Applicable. NV: not visible, V: visible; LS: lumbosacral
